# Luxatio Erecta: A Case Report and Literature Review

**DOI:** 10.7759/cureus.32976

**Published:** 2022-12-26

**Authors:** Daniel A Vidal Panduro, Elizabeth Zegarra Buitron, Alfredo B Huerta Robles

**Affiliations:** 1 Internal Medicine, School of Medicine, Universidad Peruana de Ciencias Aplicadas, Lima, PER; 2 Orthopaedics and Traumatology, Division of Trauma and Orthopaedic Surgery, SANNA - San Borja Clinic, Lima, PER

**Keywords:** hyperabduction, closed reduction, shoulder injury, inferior shoulder dislocation, luxatio erecta

## Abstract

Luxatio erecta (inferior shoulder dislocation) is a rare entity, infrequent, but with a good prognosis. There are two mechanisms for this injury to occur, by an indirect force, which is the most frequent, and by a direct force. Both involve hyperabduction of the arm. The clinical presentation is characteristic and unmistakable. Diagnosis is clinical, but imaging tests are useful to rule out associated injuries and complications. The treatment of choice is closed reduction and, in most cases with favorable results. We present the case of an 83-year-old woman who went to the emergency room with a diagnosis compatible with Luxatio erecta of the glenohumeral joint. Subsequently, a closed reduction was performed with good results. The patient is currently undergoing physical therapy and rehabilitation.

## Introduction

Luxatio erecta of the humerus or inferior dislocation of the shoulder was first described in 1859 [1]. It corresponds to an infrequent presentation injury with less than 1% of all shoulder dislocations [2], being more common in the elderly population [3]. The clinical presentation is unmistakable and the diagnosis is clinical [4]. This pathology can be associated, in great frequency, with bone, soft tissue, and neuro-vascular injuries [4]. Therefore, diagnostic support with images is recommended in order to rule out this type of lesion [5]. The treatment of choice is closed reduction due to the excellent results and good prognosis [4]. We present the case of an 83-year-old woman who, after a fall, presented a lower right shoulder dislocation. A closed reduction was performed with favorable evolution and subsequent control by an outpatient clinic.

## Case presentation

**Figure 1:**
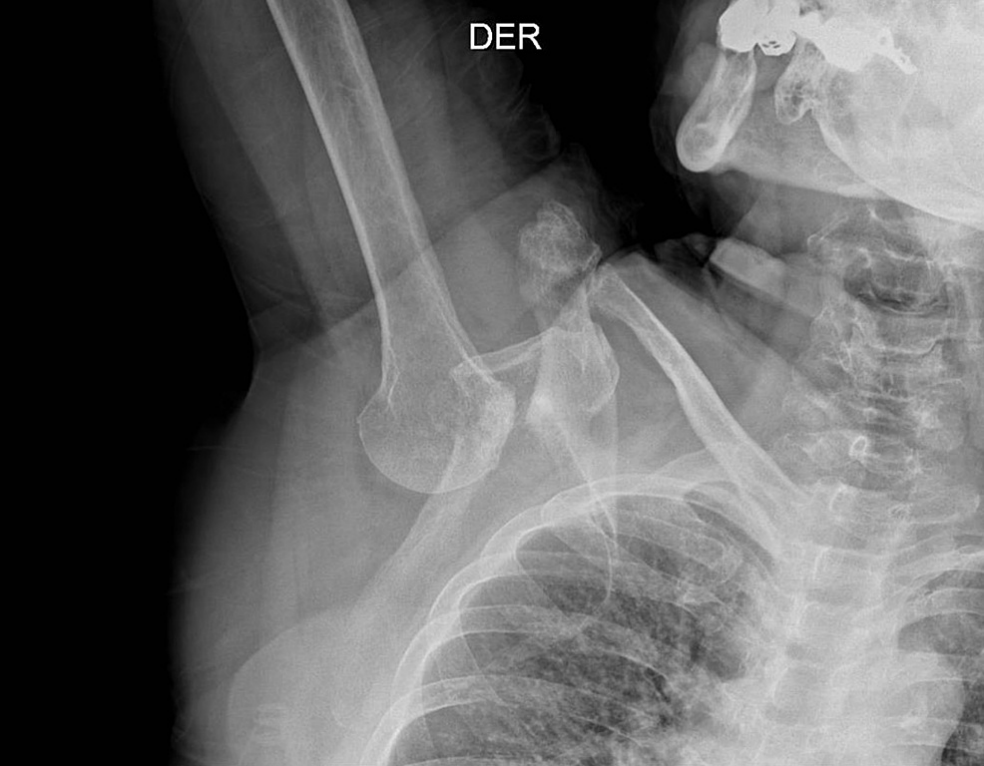
X-ray image of the right shoulder The image shows inferior glenohumeral dislocation (Luxatio erecta). There is a complete loss of glenohumeral joint congruence, with inferior displacement of the humeral head.

An 83-year-old woman with no significant history suffered a fall from her own height while she was walking. Subsequently, she presented pain and functional limitation in the right upper limb, which remained in permanent hyperabduction (at an angle of approximately 160 degrees), which is why she went to the emergency room. On physical examination, the patient was awake, complaining, and very sore, but cooperative. The right upper limb was in hyperabduction with the impossibility of adduction. No changes in skin color, sensory deficit, or any evidence of neurovascular compromise were observed. An X-ray of the right shoulder was performed, which revealed an inferior glenohumeral dislocation with no fracture signs (Figure 1).

**Figure 2:**
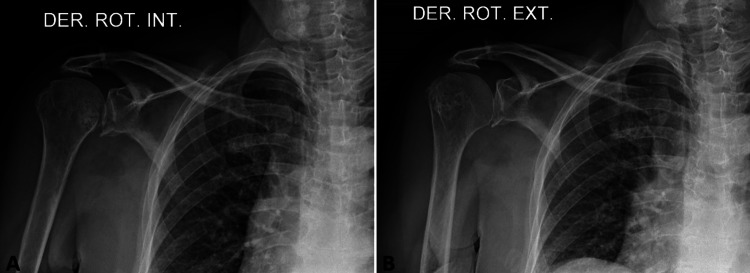
Control radiograph images after closed reduction of the dislocated joint A control radiograph was performed, which confirmed a successful reduction. (A) Internal rotation of the right shoulder. (B) External rotation of the right shoulder.

A closed reduction was carried out, after infiltration of lidocaine without epinephrine, with the Milch maneuver, followed by the Hippocrates maneuver. Subsequently, a brief adduction, hyperextension and external rotation of the right upper limb was accomplished; and joint congruence and immediate pain relief were obtained. Subsequently, a control radiograph was performed, which confirmed a successful reduction (Figure 2).

**Figure 3:**
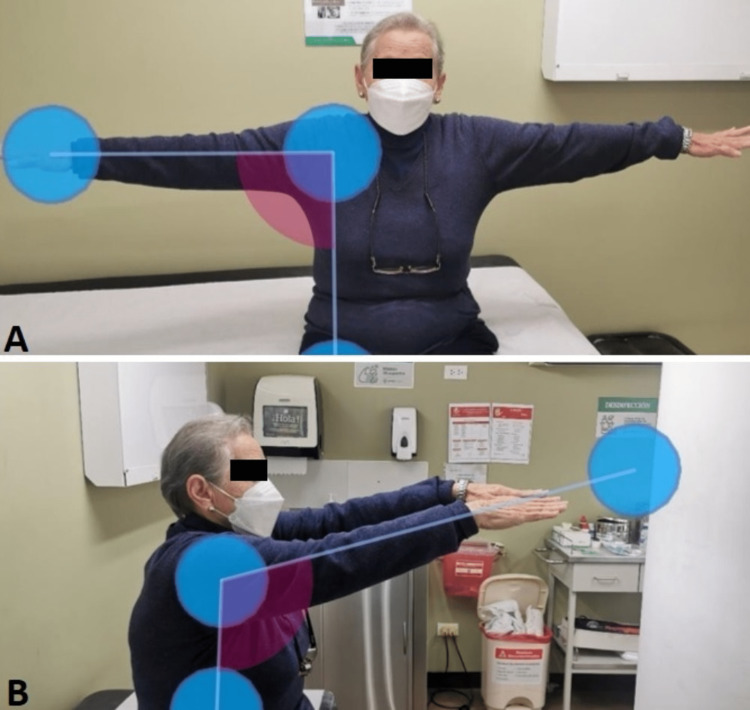
Images after six weeks of physical therapy (A) Active extension of the right shoulder: 107° (sagittal plane). (B) Abduction of the upper limb of the trunk: 90.4° (frontal plane)

**Figure 4:**
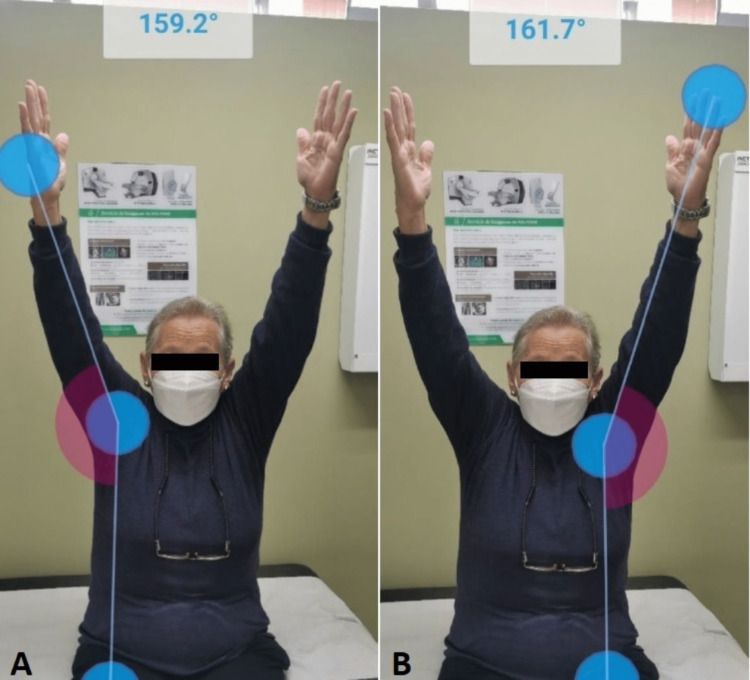
Abduction of the upper limb of the trunk after physical therapy (A) 159.2° (post dislocation) vs. (B) 161.7° (normal)

Finally, brachial, radial, and ulnar pulses were assessed with no evidence of motor or sensory deficits. The joint was immobilized in a sling for four weeks, followed by physical therapy of the right shoulder for six weeks. At control, there was evidence of improvement in joint range in flexion and abduction of the right shoulder (Figures 3, 4).

**Figure 5:**
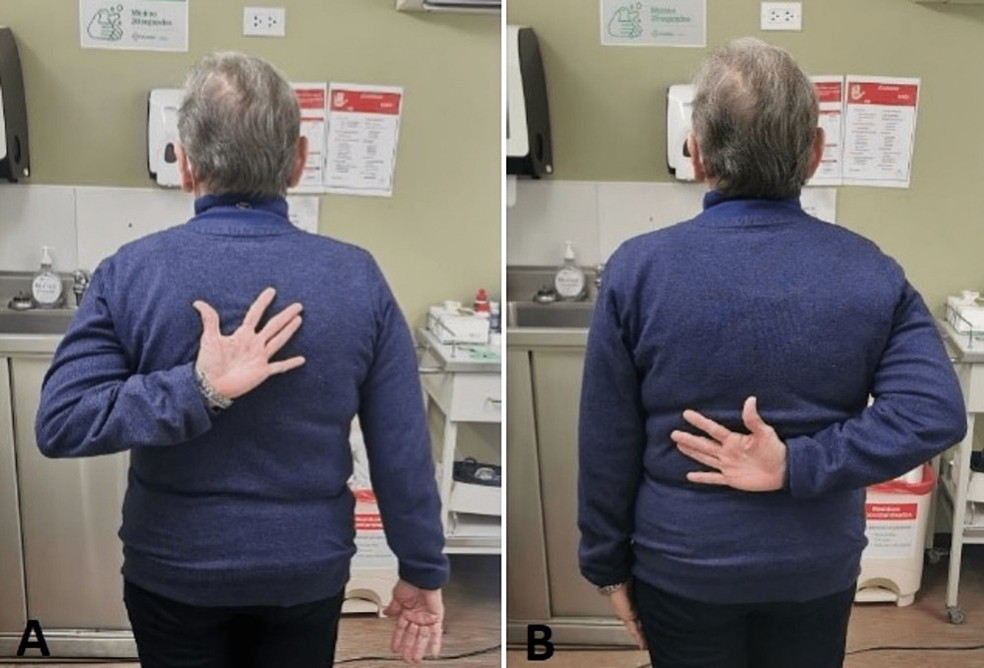
Internal rotation of the arm (A) Normal arm image. (B) Arm image after reduction of erect dislocation (six weeks later).

However, when the internal rotation of the right shoulder was performed, a marked limitation of the joint range was observed (Figure 5), so a control magnetic resonance imaging (MRI) was requested. Unfortunately, the patient did not take the exam for personal reasons. Currently, the patient continues in physical therapy. The possibility of surgical management is not ruled out according to evolution.

## Discussion

The shoulder is the joint that is most frequently dislocated due to its wide range of movement [5,6]. Shoulder dislocations are more frequently anterior 95%, and in a lower percentage posterior 4-5% and inferior 0.5% [5]. Luxatio erecta is an extremely rare and infrequent injury [2], the predisposing factors for this type of injury are advanced age and previous instability of the joint in patients with a previous history of shoulder injuries [4].

The mechanism involved in this type of injury is hyperabduction of the arm due to a violent abduction force [3], causing the neck of the humerus to straighten against the acromion, generating tearing of the inferior capsule (probably of the ligament middle and inferior glenohumeral) and mobilizing the humeral head down and out of the joint capsule [5,7].

Two mechanisms of this lesion have been described, the indirect and direct, both involve the hyperabduction of the affected arm, as in the falls from height [4]. The indirect mechanism is the most common (70%) [7,8] and is presented with shoulder hyperabduction in contact with the proximal humerus and acromion, which generates the rupture of the inferior portion of the glenohumeral capsule that leads to inferior dislocation of the humeral head [7,8]. The direct mechanism is associated with high-energy events in which the humeral head is directed down, tearing the glenohumeral ligaments [6].

The erect dislocation clinically is characterized by presenting the totally abducted arm, elevated, supported on the head, the partially flexed elbow, and the forearm in pronation [1,4,5]. This position is known as the “Hands Up” position [5,9], in some cases, it can be seen that the hand contrary to the lesion holds the affected arm in order to reduce pain [6]. Also, in thin patients, the humeral head displaced down in the axillary region can be felt [3,4,6,7].

Approximately 80% of these lesions are associated with fractures of the humerus itself or the glenoid scapular cavity, soft tissue injuries, and neuro-vascular deficits [4,5]. The fracture of the proximal humerus and the great tuberosity of the humerus are the most common, representing more than 60% of this type of injury [4]. The most common soft tissue lesions are the tear of the rotator sleeve, the capsular avulsion of the shoulder, and the disruption of the adjacent muscles [5]. More than 75% of these patients have rotator cuff lesions on MRI images [4]. Also, the most common long-term complication is adhesive capsulitis [3]. Sixty percent of these patients have some neuro-vascular compromise [9,10].

The most commonly affected structure is the axillary or circumflex nerve [3,4], followed by the brachial plexus, radial, ulnar, and median nerves [3,9] which mostly recover in a range between two weeks to one year [3]. Out of the total, 3.3% of cases present vascular compromise, with the axillary artery and the circumflex artery of the humerus being the most affected [1,3,5]. However, these injuries are mostly relieved by an early reduction of the glenohumeral joint [5].

The physical exam is essential for the diagnosis of this pathology [8]. Radiological support is necessary to confirm the diagnosis and to rule out possible complications or associated injuries [4]. That is why it is recommended to evaluate through an X​​-ray imaging prior to reduction due to the possibility of an associated fracture [5] in which you can see the humeral axis parallel to the scapular spine and the humeral head can be seen in or below the lower edge of the glenoid cavity [11,12]. On some occasions, this injury can be confused with an anterior dislocation of the shoulder, in which, radiographically, the humeral shaft is parallel to the chest wall and there is a loss of normal contour of the deltoid and a prominent acromion [11,13]. Computed tomography (CT scan) is most useful for evaluating fine fracture lines [12]. Post-reduction MRI is recommended to rule out the presence of neurovascular lesions [4]. In this, the absence of normal flow voids in the brachial, axillary, or posterior circumflex arteries could represent a vascular injury, such as thrombosis or dissection of the same [4].

The closed reduction with the traction-contraction technique, accompanied by conscious sedation, analgesia, and muscle relaxation, is the treatment of choice in most cases and this is very frequently successful [5,9]. Followed by shoulder immobilization for three to six weeks and physical therapy and rehabilitation, according to medical indication [6]. It is advised to evaluate the correct humeral position through radiography after the reduction and discard possible associated fractures [9]. Immediate reduction decreases neurovascular damage [1]. The open or surgical reduction is indicated in cases of open dislocation, fracture of the humeral head or associated lesions that require surgical treatment [4,5]. The long-term prognosis for this injury after treatment is excellent [4]. However, connective tissue structures are influenced by vascular and structural changes; increasing patient age can alter their healing ability and biomechanical function [14].

## Conclusions

Luxatio erecta of the humerus is an infrequent injury with a characteristic clinical presentation, which must be recognized by medical personnel in order to be treated promptly. This type of injury is very frequently associated with fractures, soft tissue, and neurovascular injuries, for which it is important to carry out imaging studies such as X-rays, CT scans, and MRIs. The reduction must be done early to prevent complications. For the most part, the prognosis is favorable, but age can play an important role in the healing process.
